# Prevalence of behavioral disorders and attention deficit/hyperactive disorder among school going children in Southwestern Uganda

**DOI:** 10.1186/s12888-019-2069-8

**Published:** 2019-04-03

**Authors:** Apollo Kivumbi, William Byansi, Christopher Damulira, Phionah Namatovu, James Mugisha, Ozge Sensoy Bahar, Mary M. McKay, Kimberly Hoagwood, Fred M. Ssewamala

**Affiliations:** 1 International Center for Child Health and Development, P.O. Box 1988, Circular Rd, Masaka, Uganda; 2 Brown School, Washington University in St. Louis, St. Louis, USA; 3 New York University School of Medicine, New York, USA; 4 Kyambogo University, Kampala, Uganda

**Keywords:** Prevalence, Disruptive behavior disorders, Conduct disorders, Oppositional defiant disorder, Attention deficit/hyperactive disorder, Adolescents

## Abstract

**Background:**

Disruptive Behavioral Disorders (DBDs) and Attention Deficit/Hyperactivity Disorder (ADHD) are chronic, impairing, and costly child and adolescent mental health challenges which, when untreated, can result in disruptions in school performance, friendships and family relations. Yet, there is dearth of prevalence data on child and adolescent behavioral challenges within sub-Saharan Africa, including Uganda. This study aims to estimate the prevalence rate of behavioral challenges and ADHD among young school going children and early adolescents (ages 8–13 at study enrollment), utilizing a school-based sample in southwest Uganda.

**Methods:**

We present screening results from a 5-year scale-up study titled SMART Africa-Uganda (2016–2021), set across 30 public primary schools located in the greater Masaka region in Uganda, a region heavily impacted by poverty and HIV/AIDS. Specifically, we draw on screening data from caregivers of 2434 children that used well-established standardized measures that had been pre-tested in the region. These were: 1) oppositional defiant disorder (ODD) and conduct disorder (CD) subscales of the Disruptive Behavior Disorders (DBD) scale; and 2) the Iowa Connors and Impairment scales. Slightly over half of the children in the sample were female (52%), with a mean age of 10.27 years.

**Results:**

Of the 2434 participants screened for disruptive behaviors: 1) 6% (*n* = 136) scored positive on ODD and 2% (*n* = 42) scored positive on CD subscales of the DBD scale; 2) 9.61% (*n* = 234), and 2.67% (*n* = 65) were reported to have elevated symptoms of ODD and ADHD on the Iowa Connors caregiver report scale respectively. Twenty-five percent (*n* = 586) of children were described by their caregivers as having experienced some form of impairment in at least four domains of the Impairment scale.

**Conclusion:**

The results indicate the presence of behavioral challenges and ADHD among school going children, aged 8–13 years, in Uganda. Given the negative outcomes associated with behavioral challenges as children transition to adolescence and adulthood, detecting these emerging behavioral challenges early is critical in developing appropriate interventions. School settings could be considered as one of the contextually-relevant, culturally-appropriate, and non-stigmatizing venues to implement screening procedures and to detect emerging behavioral challenges and to make necessary referrals.

## Background

Globally, Disruptive Behavioral Disorders (DBDs) are prevalent and largely contribute to the burden of diseases among children and adolescents [1, 2]. Specifically, behavioral disorders, categorized to include the following psychiatric disorders including Conduct Disorders (CD) and Oppositional Defiant Disorder (ODD) are common among children and adolescents [2]. Yet, despite being a public health problem, behavioral disorders and ADHD are still neglected conditions in primary health care in low-income countries [3], including Uganda, the focus of this paper. Behavioral disorders are characterized by signs of inattention, impulsivity, and hyperactivity, which significantly impact behavior and performance both at home and in school [3]. Research indicates that behavioral disorders are chronic, impairing, and costly mental health problems that when left untreated, result in disruptions in school performance, friendships, and family relations and, generally can affect the quality of life of children and their families [4–6].

Furthermore, DBDs and ADHD are associated with child maltreatment and abuse, and commonly co-occur alongside depression, anxiety, and substance use disorders [3]. Specifically, DBDs and ADHD are associated with academic problems, social impairment, a higher incidence of debilitating physical conditions, unemployment, legal problems, substance abuse and violence in adulthood [3, 4, 7–11]. Additionally, the impairment associated with behavioral disorders in childhood may persist through adolescence and adulthood, which places youth on a path for future school drop-out, substance use, delinquency, incarceration, criminal behaviors, and premature death [2]. Disruptive behaviors may also lead to maternal stress, which may result in poor parenting, further contributing to children’s emotional difficulties [12].

There is dearth of epidemiological data on the prevalence of behavioral disorders within sub-Saharan Africa. Though the prevalence of behavioral problems varies across cultures in the sub-Saharan Africa, estimates indicate prevalence rates ranging from 12 to 33% [8, 13, 14]. Recent estimates from a specialized children’s clinic at Mulago National Referral Hospital in Uganda reported that 11% of the participants had attention deficit hyperactive disorder (ADHD) symptoms and 8.57% had conduct disorders [15]. In a related study, Mpango et al. unraveled a prevalence rate for ADHD of 6% among children and adolescents with HIV/AIDS in specialized HIV/AIDS clinics in central and southwestern Uganda [16]. Since both studies were conducted in a clinical setting, they are likely to provide high prevalence levels for behavioral problems (given that this would be a self-selected population).

Against that backdrop, our study is the first study conducted to determine the prevalence of behavioral problems in school going children and adolescents in rural Uganda. Consequently, the current study examines: [1] rates of DBD and ADHD symptoms among school going children in rural Uganda, and [2] percentages of children described by their caregivers to experience impairment in at least four out of seven domains of the Impairment scale. Data were collected in the context of a prospective intervention study examining the effectiveness of implementation and outcomes associated with an evidence-based practice (EBP) (Hope for Families) designed to reduce childhood disruptive behavioral challenges. Specifically, the study measured ADHD, CD, ODD and the impairments associated with these disruptive disorders as reported by the children’s caregivers.

## Method

### Study design and setting

This paper utilizes data from the *SMART Africa-Uganda* scale-up study funded by the National Institute of Mental Health, (Grant #: U19MH110001). This is a longitudinal experimental study that uses a mixed methods hybrid type II effectiveness implementation design to test the effectiveness of an evidence based multiple family group intervention aimed at improving child disruptive behavioral challenges in Uganda while concurrently examining the multi-level factors that influence uptake, implementation, sustainment, and youth outcomes.

The study is set in 30 public primary schools located in five districts of the Greater Masaka region. The five geopolitical districts in southern Uganda include Masaka, Rakai, Kalungu, Lwengo and Kyotera districts. This area lies south of the equator, about 100 miles from the capital, Kampala. Historically, this region is known to have one of the highest HIV prevalence rates in Uganda at 12% compared to the national average of 7.2% [31]. This paper utilizes screening data collected between November 2017 and December 2017 from the caregivers of 2434 children on their behaviors. Children and their caregivers were recruited into the study if the child was between ages 8 to 13 years and enrolled in grades 2 through 7 from any of the participating 30 primary schools, and if both the caregiver and child consented to be in the study. In Uganda, the education system is based on seven years of primary education completed with standardized national examinations (Primary Leaving Examinations). Thus, primary 2 to 7 is equivalent to grades 2 to 7 in the U.S. system. The average age of the children in our sample is 10.27 years. The majority of participants are female (52%). Ugandan trained research assistants administered the screening tool using Qualtrics [17]. While this paper reports on screening data collected from caregivers, subsequent study assessments will include children.

### Measures and data collection

The Disruptive Behavior Disorder scale [18] has 8 items related to ODD and 15 items related to CD. Caregivers were asked to rate the severity of oppositional defiant and conduct behaviors with a scale composed of 23 items using a Likert four-point scale (1 = *not at all*, 2 = *just a little*, 3 = *pretty much*, 4 = *very much*). For example, items on the measure include *Often blames others for his or her mistakes or misbehavior –* for the ODD subscale and *Has been physically cruel to animals-*for the CD subscale. Items of the sub-scales were summed to compute presence of DBDs (range 8–32 and 15–60 for ODD and CD sub-scales respectively). This study utilized the ODD and CD subscales reported at screening. The sub-scales had α = 0.78 and α = 0.729 for ODD and CD respectively. These are considered acceptable levels. Respondents were considered positive for ODD if they endorsed a total of 4 or more items as “pretty much” or “very much”. Similarly, respondents were considered positive for CD if they endorsed a total of 3 or more items in any category or any combination of categories as “pretty much” or “very much” on items 9 through 23.

The Iowa Connors scale [19] is a short measure widely used to assess the severity of inattentive-impulsive-overactive (ADHD) and ODD behavior among children [19]. The scale is composed of 10 items using a Likert scale (0 = *not at all*, 1 = *just a little*, 2 = *pretty much*,3 = *very much)*. Examples of questions in the scale are *Temper outburst – behavior explosive and unpredictable*-for ODD subscale and *Inattentive, easily distracted*-for ADHD subscale. Items 1 through 5 measure ADHD and items 6 through 10 measure ODD respectively. Completed by the child’s parent/caregiver, items of the sub-scales were summed to compute for presence of DBDs (range 0–15 for each sub-scale). This study utilized the ODD and ADHD subscales reported at screening (α = 0.697 and α = 0.612 respectively). These are considered moderate levels. Respondents were considered positive for ADHD if they scored 9 or higher for questions 1 through 5. In the same way, respondents were considered positive for ODD if they scored 9 or higher for questions 6 through 10.

For the Impairment scale [20], caregivers were asked to rate the severity of the impact of their children’s difficulties across functional domains on this 6-item rating scale (e.g., relationship with playmates or peers, relationship with the parent(s), relationship with sibling(s), academic progress, family functioning, self-esteem). The visual analogue scale ranges from 1 (*Not a problem at all*; definitely does not need treatment or special services) to 6 (*Extreme problem*; definitely needs treatment and special services), with higher scores indicating greater impairment. For the current study, we utilized a summed score of the impairment items measured at screening. A score of 3 or higher in four or more domains was considered a significant impairment.

### Ethics and study procedures

The study obtained ethics approval from Washington University in St. Louis (approval number:201611088), Uganda National Council of Science and Technology (SS4090), Uganda Virus Research Institute (GC/127/17/06/555), as well as the Data Safety and Monitoring Board at NIMH. Both caregivers and children were explained the aim and objectives of the study. Caregivers provided written consent for their children to participate in the study in addition to their own participation and all children were assented. All research assistants completed the National Institutes of Health's (NIH) Good Clinical Practice and CITI Human Subjects trainings. All measures were translated from English to Luganda and back translated. All data collected were kept under key and lock and only accessible to the research team.

### Data analysis

We conducted univariate analyses—to describe the sample; bivariate analyses to understand behavioral disorders by gender, and chi-square to assess the statistical significance of the predominately reported/observable behavioral disorders. The data were analyzed using Stata software SE. 12.1.

## Results

From Table 1, the mean age of the sample was 10.27 years with males being significantly older (.0001) than their female counterparts (10.53 vs 10.03). Most of the study schools were located in the semi urban regions of southwestern Uganda with their population accounting for 45.07% while schools in urban settings accounted for 11.05%.

**Table 1:** Sample characteristics (*N* = 2434)

Variable	Total n(%)	Male n(%)	Female n(%)	*p*-value	χ and t-value
Age: years Mean (SD)	10.27 (1.35)	10.53 (1.32)	10.03 (1.33)	0.0000	9.18***
Location of the school				0.046	6.16*
Rural	1068 (43.88)	492 (42.27)	576 (45.35)		
Semi-urban	1097 (45.07)	554 (47.59)	543 (42.76)		
Urban	269 (11.05)	118 (10.14)	151 (11.89)		

*P ≤ 0.05; *** *P* ≤ 0.0001

Table 2 presents frequencies and percentages of prevalence rates by gender. Overall, of the 2434 participants screened for disruptive behaviors, 151 (6.2%) evidenced DBDs, of which, 84 (6.61%) and 67 (5.76%) of females and males scored positive on DBD scale respectively. In addition, 136 (6%) scored positive on ODD and 42 (2%) scored positive on CD subscales of the DBD scale.

**Table 2:** Behavioral Disorders across gender (*N* = 2434)

	Positive n (%)			Negative (%, n)					Chi-square (χ^2)^
	Male	Female	Total	Male	Female	Total	*p*-value	df	
Disruptive Behavioral Disorder (DBD)	67 (5.76)	84 (6.61)	151 (6.20)	1097 (94.24)	1186 (93.39)	2283 (93.80)	0.381	1	0.769
Oppositional Defiant Disorder	60 (5.15)	76 (5.98)	136 (5.59)	1104 (94.85)	1194 (94.02)	2298 (94.41)	0.373	1	0.792
Conduct Disorder	19 (1.63)	23 (1.81)	42 (1.73)	1145 (98.37)	1247 (98.19)	2392 (98.27)	0.735	1	0.114
Iowa Connors Scale	115 (9.88)	153 (12.05)	268 (11.01)	1049 (90.12)	1117 (87.95)	2166 (88.99)			
ADHD	29 (2.49)	36 (2.83)	65 (2.67)	1135 (97.51)	1234 (97.17)	2369 (97.33)			
ODD	97 (8.33)	137 (10.79)	234 (9.61)	1067 (91.67)	1133 (89.21)	2200 (90.39)	0.040	1	4.209*
Impairment Scale [With at least 4-items]	289 (24.83)	297 (23.39)	586 (24.08)	875 (75.17)	973 (76.61)	1848 (75.92)	0.406	1	0.691

On the ODD sub-scale, approximately 6 % of the participants evidenced symptoms of ODD, of which, 76 (5.98%) females and 60 (5.15%) males evidenced ODD symptoms. Similarly, approximately 2 % of participants in our study sample evidenced symptoms of CD. Of these, 23 (1.81%) are females while 19 (1.63%) are males.

Additionally, approximately 268 (11.01%) of the participants scored positive on the Iowa Connors Scale (**χ**^**2**^ **=** 4.209; *p* ≤ .05). Of these, 153 (12.05%) and 115 (9.88%) of females and males scored positive on Iowa Connors Scale respectively. In addition, 65 (2.67%) scored positive on ADHD and 234 (9.61%) scored positive on ODD subscales of the Iowa Connors scale. Finally, for 586 (25%) of the children, caregivers expressed concern in at least four impairment domains. Of the positives on the scale, the majority of the participants are male (24.83%). Generally as seen in Tables 3 and 4, DBDs and ADHD were found to be more prevalent in the urban regions and among 11–13 year old children compared to semi-urban, rural and 8–10 year old children, although these distributions were not statistically significant.

**Table 3:** Behavioral Disorders across different age group (N = 2434)

Variables	Positive (%, n)			Negative (%, n)			*p*-value	df	Chi-square (χ2)
	8 to 10 YEARS	11 to 13 YEARS	Total	8 to 10 YEARS	11 to 13 YEARS	Total			
Disruptive Behavioral Disorder (DBD)	6.03 (87)	6.45 (64)	6.20 (151)	93.97 (1355)	93.55 (925)	93.80 (2283)	0.674	1	0.177
Oppositional Defiant Disorder	5.48 (79)	5.75 (57)	5.59 (136)	94.52 (1363)	94.25 (935)	94.41 (2298)	0.778	1	0.079
Conduct Disorder	1.66 (24)	1.81 (18)	1.73 (42)	98.34 (1418)	98.19 (974)	98.27 (2392)	0.780	1	0.078
Iowa Connors Scale	10.47 (151)	11.79 (117)	11.01 (268)	89.53 (1291)	88.21 (875)	88.99 (2166)	0.306	1	1.049
ADHD	2.29 (33)	3.23 (32)	2.67 (65)	97.71 (1409)	96.77 (960)	97.33 (2369)	0.159	1	1.987
ODD	9.43 (136)	9.88 (98)	9.61 (234)	90.57 (1306)	90.12 (894)	90.39 (2200)	0.713	1	0.136
Impairment Scale [With at least 4-items]	23.65 (341)	24.70 (245)	24.08 (586)	76.35 (1101)	75.30 (747)	75.92 (1848)	0.552	1	0.354

*P* ≤ 0.05

**Table 4:** Behavioral Disorders across different school location (N = 2434)

	Positive (%, n)				Negative (%, n)				df	*P* - Values	Chi-square (χ2)
	Rural	Semi-urban	Urban	Total	Rural	Semi-urban	Urban	Total			
Disruptive Behavioral Disorder (DBD)	5.43 (58)	6.75 (74)	7.06 (19)	6.20 (151)	94.57 (1010)	93.25 (1023)	92.94 (250)	93.80 (2283)	2	0.369	1.992
Oppositional Defiant Disorder	4.78 (51)	6.11 (67)	6.69 (18)	5.59 (136)	95.22 (1017)	93.89 (1030)	93.31 (251)	94.41 (2298)	2	0.284	2.519
Conduct Disorder	1.5 (16)	1.82 (20)	2.23 (6)	1.73 (42)	98.50 (1052)	98.18 (1077)	97.77 (263)	98.27 (2392)	2	0.673	0.792
Iowa Connors Scale	9.83 (105)	12.22 (134)	10.78 (29)	11.01 (268)	90.17 (963)	87.78 (963)	98.22 (240)	88.99 (2166)	2	0.207	3.154
ADHD	1.87 (20)	3.28 (36)	3..35 (9)	2.67 (65)	98.13 (1048)	96.72 (1061)	96.65 (260)	97.33 (2369)	2	0.097	4.664
ODD	8.61 (92)	10.57 (116)	9.67 (26)	9.61 (234)	91.39 (976)	89.43 (981)	90.33 (243)	90.39 (2200)	2	0.302	2.394
Impairment Scale [With at least 4-items]	23.69 (253)	24.25 (266)	24.91 (67)	24.08 (586)	76.31 (815)	75.75 (831)	75.09 (202)	75.92 (1848)	2	0.902	0.207

## Discussion

To our knowledge, this is the first study conducted to determine the prevalence of behavioral problems among school going children and adolescents in rural Uganda. Data from multiple measures demonstrate the existence of behavioral problems among school children. Specifically, the 9.61 and 5.59% estimated prevalence of ODD as measured by Iowa Connors scale and DBD scale respectively were high. Similarly, approximately 2 % of the sample evidenced symptoms of CD measured by the DBD scale. Moreover, 2.67% screened positive on ADHD using the Iowa Connors scale. Several studies conducted in other sub-Saharan countries have reported prevalence rates that range between 1 to 20% [1]. For instance, in South Africa, Bakare reported that the prevalence of behavioral problems among school going children, particularly ADHD, varies between 5.4 to 8.7% [21], while Chinawa et al. reported a prevalence rate of 3% for ADHD among school going children seeking treatment at health facilities in Nigeria [22]. Our prevalence rates for DBDs as measured by DBD scale (6.2%) and Iowa Connors scale (11%) are not different. Not many studies have looked at DBDs like our study. Most studies conducted in Uganda have focused on ADHD and been carried out in health facilities. For example, Wamulugwa et al. reported a prevalence rate of 11% and Mpango et al. reported a prevalence rate of 6% for ADHD among children with psychiatric diagnoses and HIV-positive adolescents respectively in Uganda [15, 16]. Our study reported lower rates of ADHD (2.67%), which could be most likely due to the difference in study population. Populations used in these studies were clinical samples, unlike our community-based sample.

Similarly, another study conducted in Ethiopia among a community sample found the ADHD prevalence to be at 1.5%, which is consistent with our findings (2.67%) [23]. Wamulugwa and colleagues found prevalence rates of 11% for ADHD and 8.5% for CD among children with neurological and psychiatric disorders [15], yet our study was done in rural public schools among children who had no prior neurological or psychiatric diagnoses. On the other hand, prevalence rates of ADHD in studies conducted in the United States (US) among national samples [24, 25] are also in range with prevalence rates reported in clinical samples in sub-Saharan Africa. For instance, among a sample of children aged 4–17 years in the US, the study found prevalence rate of 11% for ADHD [24], consistent with the results reported by Wamulugwa and colleagues among a clinical sample [15]. one should note that the overdiagnosis of ADHD has been pointed out as a concern in the United States [25]. Hence, the variation observed could be largely attributed to the different age groups included and/or the use of clinical versus community samples. Thus, our prevalence rate is consistent with other study findings with community samples in sub-Saharan Africa and may be a true reflection of the prevalence of behavioral problems among school children.

In addition, 25% of children were described by their caregivers to experience impairment in at least four out of six domains of the Impairment scale. As a result, the prevalence rates reported in this study for DBDs could potentially impact school performance, social functioning as well as peer interaction among children and requires immediate public health interventions. The high reported rates of impairment associated with behavioral problems could be attributed to abject poverty, violence and parental mortality and HIV/AIDS [16, 22].

Our study contributes to scientific knowledge by providing epidemiological data on the prevalence of DBDs among school-going children in Uganda. Our results indicate a high prevalence of DBDs among children, which are associated with negative health and educational outcomes including delinquency, violence, drug use, poor academic performance, and anti-social personality if left untreated. Given the negative developmental outcomes associated with DBDs, particularly on academic performance and social functioning [26, 27], it is essential to screen and identify DBDs at their onset and provide necessary mental healthcare. Assessment and diagnosis are necessary in early identification of the disorders and in determining how to target and tailor interventions for specific disorders. Additionally, it is paramount to establish and develop robust context specific surveillance systems that facilitate screening, treatment and monitoring of children with DBDs.

Secondly, our results have vital implications for programming and policy, especially in sub-Saharan Africa where in most cases, there is severe scarcity of mental health professionals, evidence based interventions as well as mental health policy guidelines. The findings underscore the need to provide child and adolescent mental health training to school teachers to equip them with skills necessary for screening and early identification of DBDs and ADHD. These can include short-term certificates, as well as advanced degree programs tailored for school professionals with a potential to expand child and adolescent mental health care workforce. This calls for training not only in identification but also training in the delivery of brief, low-cost and effective evidence-based interventions developed and−/or adapted to the Uganda/sub-Saharan Africa context. In addition, there is need to support and provide ongoing training and supervision to community health workers/lay workers that already exist in the health and education systems. Previous research has underscored the positive mental health and psychosocial outcomes from interventions delivered by lay workers with little or no prior mental health training [28]. In the Uganda context, these would include teachers, community health workers, village health teams as well as parent-teacher association members that work directly with students and within communities. Further, there is a need to build and strengthen the mental health policy framework in sub-Saharan Africa which explicitly addresses strengthening mental health care needs in non-stigmatizing settings including families, schools and primary health care clinics.

The findings presented in this study require careful interpretation in light of the following limitations. First, although the study was conducted in many schools, we cannot conclude that rates of DBDs evidenced in the sample are nationally representative of school going children. Moreover, study findings are based on children in rural Uganda, enrolled in grades 2 through 7, between the ages of 8–13- years-old as specified in our inclusion criteria. As a result, findings are not necessarily generalizable to families with older or younger children in both rural and urban areas. Certainly, the large sample of children involved in this study provides reliable estimates of the prevalence of DBDs among rural school children in Uganda.

Secondly, this study exclusively relied on parent reports of behavior for measures of DBDs. Understandably, having multiple reporters (e.g., parents, teachers, non-teaching staff and children) would have provided additional information on the symptoms exhibited by children. This is a notable limitation of the current study. However, other studies have relied on caregiver reports using similar measures which increase confidence in the validity of the prevalence rates found in this study [15, 29, 30].

## Conclusion

Our study found a high occurrence of symptoms of behavioral problems as reported by children’s caregivers. Based on caregiver reports, we find relatively high occurrences of child behavioral problems in the greater Masaka community due to the region having a high prevalence to HIV/AIDs and high rates of parental death from the epidemic [31]. School interventions have been deemed successful in the treatment of other mental health disorders. With that in mind, it is vital to develop effective care and interventions for school going children. In addition, there is a need for urgent care of children experiencing behavioral difficulties since untreated behavioral problems are associated with huge psychological difficulties at the individual, family and community levels.

## Abbreviations

ADHD: Attention deficit/hyperactive disorder
CD: Conduct disorder
DBDs: Disruptive behavior disorders
NIH: National Institutes of health
NIMH: National Institute of mental health
ODD: Oppositional defiant disorder
SMART Africa: Strengthening Mental Health and Research Training in Africa
US: United States
